# Supplementary material for: Nucleus accumbens circuit disinhibits lateral hypothalamus glutamatergic neurons contributing to morphine withdrawal memory in male mice
Source: Nat Commun. 2023 Jan 5;14:71. doi: 10.1038/s41467-022-35758-5 (PMC9814415; doi:10.1038/s41467-022-35758-5)
Supplement: Supplementary file 1 — Supplementary Information [file 41467_2022_35758_MOESM1_ESM.pdf]

Nucleus accumbens circuit disinhibits lateral hypothalamus glutamatergic neurons contributing to morphine withdrawal memory in male mice

Huan Sheng*[1, 2], Chao Lei*[1], Yu Yuan*[1], Yali Fu[1], Dongyang Cui[1], Li Yang[1], Da Shao[1], Zixuan Cao[1], Hao Yang[1], Xinli Guo[1], Chenshan Chu[1], Yaxian Wen[1], Zhangyin Cai[1], Ming Chen[#1], Bin Lai[#1], Ping Zheng[#1,3]

[1]State Key Laboratory of Medical Neurobiology, Institutes of Brain Science, MOE Frontier Center for Brain Science, Department of Neurology of Zhongshan Hospital, Fudan University, Shanghai 200032, China

[2]Department of Otorhinolaryngology-Head and Neck Surgery, Zhongshan Hospital, Fudan University, Shanghai, 200032, China

[3]Medical College of China Three Gorges University, Yichang 443002, China

* These authors contributed equally to this work.

[#]Correspondence author email address: Ping Zheng: pzheng@shmu.edu.cn; Bin Lai: laibin@fudan.edu.cn or Ming Chen: 09111010024@fudan.edu.cn.

Figure S1

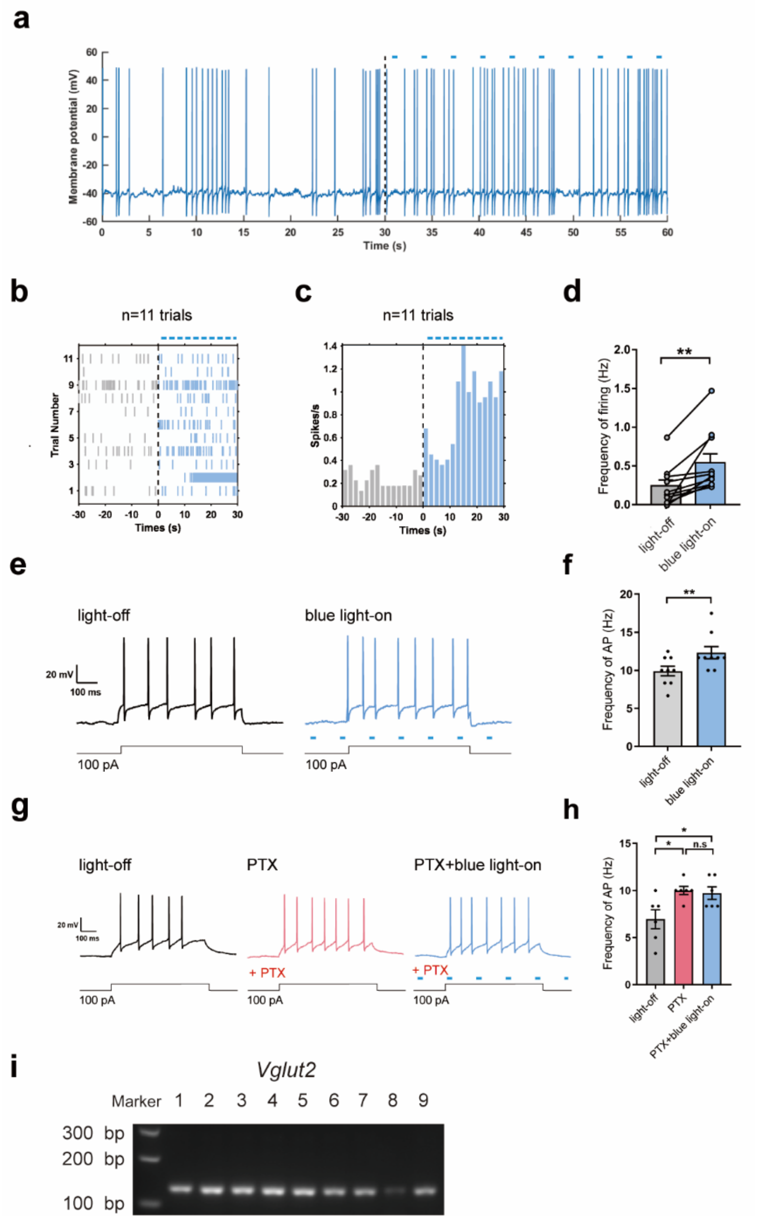

Fig. S1 The infulence of optogenetic activation of D1-MSNs projecting from NAcC to LH on the activity of LH glutamatergic neurons.

a Representative spontaneous firing traces before and after blue light stimulation in the fluorescent labeled LH glutamatergic neurons. **b** Raster plot of the 11 cells of fluorescent labeled LH glutamatergic neurons in response to a blue light stimulation. **c** PSTH of the 11 cells of fluorescent labeled LH glutamatergic neurons in response to a blue light stimulation. **d** Average frequency of spontaneous firing before and after blue

light stimulation (n = 11 cells). Paired t test, $p = 0.0038$. **e** The representative AP in non-fluorescent labeled LH glutamatergic neurons in the light-off and blue light-on groups. **f** The average frequency of AP in non-fluorescent labeled LH glutamatergic neurons in the light-off and blue light-on groups (n = 9 cells). Paired t test, $p = 0.0027$. **g** The representative AP in non-fluorescent labeled LH glutamatergic neurons by the current or before and after the light stimulation (blue light, 470 nm, 2 ms) in the presence of PTX. **h** The average frequency of AP in non-fluorescent labeled LH glutamatergic neurons in the light-off, PTX and PTX + blue light-on group (n = 6 cells). One-way ANOVA, $F_{(5, 10)} = 5.067$, $p = 0.0142$. Tukey's multiple comparisons: light-off group vs. PTX group: $p = 0.0438$，light-off group vs. PTX + blue light-on group: $p = 0.0094$, PTX group vs. PTX + blue light-on group: $p = 0.8547$. **i** Single-cell RT–PCR from the recorded neurons demonstrated that non-fluorescent labeled LH neurons expressed *Vglut2*. Mean ± SEMs. * $p < 0.05$, ** $p < 0.01$.

Table 1. The number of eliminated mice due to the inaccurate injection site in each experiment

Experimental program	Mouse strain	Injection site	Number of mice (stereotactic surgery)	Number of statistical mice	Number of eliminated mice
chemogenetic inhibition of NAcC D1-MSNs (Fig. 2c)	*D1-cre*	NAcC	22	20	2
chemogenetic inhibition of NAcC D2-MSNs (Fig. 2e)	*D2-cre*	NAcC	18	17	1
chemogenetic inhibition of NAcC-LH D1 projection neurons (Fig. 3i)	*D1-cre*	NAcC and LH	34	31	3
chemogenetic inhibition of LH glutamatergic neurons (Fig. 1g)	*Vglut2-cre*	LH	32	30	2
retrograde labeled NAcC-LH D1 projection neurons (Fig. 3c)	C57BL/6J	LH	26	24	2
PPR patch recording (Fig. 5f)	*D1-cre*	NAcC	16	16	0
IPSC patch recording (Fig. 5b and Fig. 5c)	*D1-Cre::Vglut2-Flpo*	NAcC and LH	10	10	0
IPSC patch recording (Fig. 6b)	*Vglut2-cre*	LH	6	5	1
AP patch recording (Fig. 3e , Fig. 3g and Fig. 6e)	*D1-Cre::Vglut2-Flpo*	NAcC and LH	16	16	0
