# Supplementary material for: Nucleus accumbens circuit disinhibits lateral hypothalamus glutamatergic neurons contributing to morphine withdrawal memory in male mice
Source: Nat Commun. 2023 Jan 5;14:71. doi: 10.1038/s41467-022-35758-5 (PMC9814415; doi:10.1038/s41467-022-35758-5)
Supplement: Supplementary file 3 — Reporting Summary [file 41467_2022_35758_MOESM3_ESM.pdf]

nature portfolio

Corresponding author(s): Ping Zheng

Last updated by author(s): Dec 12, 2022

Reporting Summary

Nature Portfolio wishes to improve the reproducibility of the work that we publish. This form provides structure for consistency and transparency in reporting. For further information on Nature Portfolio policies, see our Editorial Policies and the Editorial Policy Checklist.

Statistics

For all statistical analyses, confirm that the following items are present in the figure legend, table legend, main text, or Methods section.

n/a	Confirmed	
☐	☒	The exact sample size (*n*) for each experimental group/condition, given as a discrete number and unit of measurement
☐	☒	A statement on whether measurements were taken from distinct samples or whether the same sample was measured repeatedly
☐	☒	The statistical test(s) used AND whether they are one- or two-sided<br>*Only common tests should be described solely by name; describe more complex techniques in the Methods section.*
☒	☐	A description of all covariates tested
☐	☒	A description of any assumptions or corrections, such as tests of normality and adjustment for multiple comparisons
☐	☒	A full description of the statistical parameters including central tendency (e.g. means) or other basic estimates (e.g. regression coefficient) AND variation (e.g. standard deviation) or associated estimates of uncertainty (e.g. confidence intervals)
☐	☒	For null hypothesis testing, the test statistic (e.g. *F*, *t*, *r*) with confidence intervals, effect sizes, degrees of freedom and *P* value noted<br>*Give P values as exact values whenever suitable.*
☒	☐	For Bayesian analysis, information on the choice of priors and Markov chain Monte Carlo settings
☒	☐	For hierarchical and complex designs, identification of the appropriate level for tests and full reporting of outcomes
☒	☐	Estimates of effect sizes (e.g. Cohen's *d*, Pearson's *r*), indicating how they were calculated

Our web collection on statistics for biologists contains articles on many of the points above.

Software and code

Policy information about availability of computer code

Data collection	Behavior data: Three Compartment Place Preference (Med Associates, USA) , Whole cell recording: PatchMaster 2.54 software (HEKA).
Data analysis	Graphpad Prism 8.4, Mini anlysis, Image J and Adobe Photoshop CC 2018.

For manuscripts utilizing custom algorithms or software that are central to the research but not yet described in published literature, software must be made available to editors and reviewers. We strongly encourage code deposition in a community repository (e.g. GitHub). See the Nature Portfolio guidelines for submitting code & software for further information.

Data

Policy information about availability of data

All manuscripts must include a data availability statement. This statement should provide the following information, where applicable:

- Accession codes, unique identifiers, or web links for publicly available datasets
- A description of any restrictions on data availability
- For clinical datasets or third party data, please ensure that the statement adheres to our policy

All data supporting the study are provided within the main text and extended data.

Field-specific reporting

Please select the one below that is the best fit for your research. If you are not sure, read the appropriate sections before making your selection.

☒ Life sciences ☐ Behavioural & social sciences ☐ Ecological, evolutionary & environmental sciences

For a reference copy of the document with all sections, see nature.com/documents/nr-reporting-summary-flat.pdf

Life sciences study design

All studies must disclose on these points even when the disclosure is negative.

Sample size	Sample sizes were determined by consulting existing literature using similar techniques and were kept to a minimum to be compliant with local animal usage policies.
Data exclusions	The mice with a strong preference (> 80%) for any compartment on pre-test of CPA or incorrect viral injection sites were excluded.
Replication	For behavioural experiments all reported n come from separate animals. And all data related behavior were collected from more than one batch mice. And all the histological experiments were collected from at least 3 mice.
Randomization	All animal were randomly assigned to different groups.
Blinding	All behavior related data were performed, collected and analyzed blindly.

Reporting for specific materials, systems and methods

We require information from authors about some types of materials, experimental systems and methods used in many studies. Here, indicate whether each material, system or method listed is relevant to your study. If you are not sure if a list item applies to your research, read the appropriate section before selecting a response.

Materials & experimental systems

n/a	Involved in the study	
☐	☒	Antibodies
☒	☐	Eukaryotic cell lines
☒	☐	Palaeontology and archaeology
☐	☒	Animals and other organisms
☒	☐	Human research participants
☒	☐	Clinical data
☒	☐	Dual use research of concern

Methods

n/a	Involved in the study	
☒	☐	ChIP-seq
☒	☐	Flow cytometry
☒	☐	MRI-based neuroimaging

Antibodies

Antibodies used	guinea pig anti–c-Fos antibody: Synaptic Systems, Cat# 226004, dilution 1:1000;<br>goat anti-guinea pig immunoglobulin G (IgG) antibody: Vector, Cat# BA-7000, dilution 1:500;<br>Streptavidin-Cy3: Sigma-Aldrich, Cat# S6402, dilution 1:1000.
Validation	The guinea pig anti–c-Fos antibody as primary antibody has been widely used. According to the website (https://www.sysy.com/product/226004#list).

Animals and other organisms

Policy information about studies involving animals; ARRIVE guidelines recommended for reporting animal research

Laboratory animals	All mice used in the study were adult males aged between 8-12 weeks. including C57BL/6J and transgenic mice.
Wild animals	No wild animals were used.
Field-collected samples	No field-collected samples were used.
Ethics oversight	All experimental procedures conformed to Fudan University as well as international guidelines on the ethical use of animals.

Note that full information on the approval of the study protocol must also be provided in the manuscript.
